# Colchicine modulates calcium homeostasis and electrical property of HL‐1 cells

**DOI:** 10.1111/jcmm.12818

**Published:** 2016-02-29

**Authors:** Yen‐Yu Lu, Yao‐Chang Chen, Yu‐Hsun Kao, Yung‐Kuo Lin, Yung‐Hsin Yeh, Shih‐Ann Chen, Yi‐Jen Chen

**Affiliations:** ^1^ Division of CardiologySijhih Cathay General HospitalNew Taipei CityTaiwan; ^2^ School of MedicineFu‐Jen Catholic UniversityNew Taipei CityTaiwan; ^3^ Department of Biomedical EngineeringNational Defense Medical CenterTaipeiTaiwan; ^4^ Graduate Institute of Clinical MedicineCollege of MedicineTaipei Medical UniversityTaipeiTaiwan; ^5^ Department of Medical Education and ResearchWan Fang HospitalTaipei Medical UniversityTaipeiTaiwan; ^6^ Cardiovascular DivisionChang Gung Memorial HospitalChang Gung University College of MedicineTaoyuanTaiwan; ^7^ School of MedicineNational Yang‐Ming UniversityTaipeiTaiwan; ^8^ Division of Cardiology and Cardiovascular Research CenterVeterans General Hospital‐TaipeiTaipeiTaiwan; ^9^ Division of Cardiovascular MedicineDepartment of Internal MedicineWan Fang HospitalTaipei Medical UniversityTaipeiTaiwan

**Keywords:** colchicine, calcium handling, electrophysiology

## Abstract

Colchicine is a microtubule disruptor that reduces the occurrence of atrial fibrillation (AF) after an operation or ablation. However, knowledge of the effects of colchicine on atrial myocytes is limited. The aim of this study was to determine if colchicine can regulate calcium (Ca^2+^) homeostasis and attenuate the electrical effects of the extracellular matrix on atrial myocytes. Whole‐cell clamp, confocal microscopy with fluorescence, and western blotting were used to evaluate the action potential and ionic currents of HL‐1 cells treated with and without (control) colchicine (3 nM) for 24 hrs. Compared with control cells, colchicine‐treated HL‐1 cells had a longer action potential duration with smaller intracellular Ca^2+^ transients and sarcoplasmic reticulum (SR) Ca^2+^ content by 10% and 47%, respectively. Colchicine‐treated HL‐1 cells showed a smaller L‐type Ca^2+^ current, reverse mode sodium–calcium exchanger (NCX) current and transient outward potassium current than control cells, but had a similar ultra‐rapid activating outward potassium current and apamin‐sensitive small‐conductance Ca^2+^‐activated potassium current compared with control cells. Colchicine‐treated HL‐1 cells expressed less SERCA2a, total, Thr17‐phosphorylated phospholamban, Cav1.2, CaMKII, NCX, Kv1.4 and Kv1.5, but they expressed similar levels of the ryanodine receptor, Ser16‐phosphorylated phospholamban and Kv4.2. Colchicine attenuated the shortening of the collagen‐induced action potential duration in HL‐1 cells. These findings suggest that colchicine modulates the atrial electrical activity and Ca^2+^ regulation and attenuates the electrical effects of collagen, which may contribute to its anti‐AF activity.

## Introduction

Atrial fibrillation (AF) is the commonest form of sustained arrhythmia and increases the risk of stroke, heart failure and mortality 1, 2. However, AF rhythm control agents are not very useful due to a high recurrence of AF, low adherence and common adverse effects 3, 4. Colchicine, a drug widely used to treat gouty arthritis, was found to reduce the occurrence of post‐operative AF or early AF recurrence after pulmonary vein isolation in the absence of antiarrhythmic drug treatment 5, 6. Colchicine has a potent anti‐inflammatory effect and decreases the occurrence of pericarditis 7, 8, 9, both critical in the pathogenesis of AF. Moreover, colchicine can directly modulate calcium (Ca^2+^) homeostasis in cardiomyocytes 10. However, it is not clear if colchicine can regulate atrial electrical activity to reduce AF genesis 11.

Colchicine is a microtubule disruptor that inhibits microtubule assembly in cells and inhibits neutrophil and endothelial cell adhesion molecules 5, 12, which play roles in the pathophysiology of AF 13. Microtubules, a major cytoskeletal component of cardiomyocytes, are linked to various pathological conditions and may play a role in modulating both the electrical and mechanical activities of the heart. Moreover, microtubule assembly increases the deposition of extracellular matrix (ECM) proteins, especially type I collagen 14, 15. Higher concentrations of ECM proteins enhance the occurrence of AF through electrical and structural remodelling 16, 17. Colchicine decreases interstitial myocardial fibrosis by interfering with collagen accumulation and reverses the contractile function in failing hearts 18, 19. Therefore, colchicine may attenuate myocardial remodelling through decreasing ECM accumulation and prevent AF occurrence. The purpose of this study was to investigate the effects of colchicine on Ca^2+^ homeostasis and electrophysiological characteristics in atrial myocytes and find out if colchicine can modulate collagen‐induced action potential (AP) changes.

## Materials and methods

### Cell culture

HL‐1 cells derived from mouse atrial cardiac muscle cells (kindly provided by Dr. Claycomb, Louisiana State University Health Sciences Center, New Orleans, LA, USA) were cultured in a humidified atmosphere of 5% CO_2_ at 37°C in Claycomb medium (JRH Biosciences, Lenexa, KS, USA). HL‐1 cells were seeded in fibronectin/gelatin‐precoated dishes as described previously. Cells were plated at a density of 5 × 10^5^ cells/well in 6‐well culture plates. At 24 hrs after cell seeding, we added colchicine (3 nM, which is within the therapeutic concentration) 20 or a vehicle solution to cells for a further 24 hrs as colchicine‐treated or control cells, respectively. We also studied the effect of colchicine on collagen‐treated HL‐1 cells. We incubated HL‐1 cells with collagen (10 mg/ml) and colchicine (3 nM) for a further 24 hrs after cell seeding.

### Measurement of intracellular Ca^2+^

As described previously 21, HL‐1 cells were loaded with fluorescent Ca^2+^ (10 μmol/l) fluo‐3/acetoxymethyl (AM) ester for 30 min. at room temperature. Excess extracellular dye was removed by changing the bath solution, and intracellular hydrolysis of fluo‐3/AM occurred at 35 ± 1°C after 30 min. Fluo‐3 fluorescence was excited with a 488‐nm line of an argon ion laser. The emission was recorded at >515 nm. Cells were repetitively scanned at 3‐ms intervals for a total duration of 6 sec. Fluorescence imaging was performed with a laser scanning confocal microscope (Zeiss LSM 510, Carl Zeiss, Jena, Germany) and an inverted microscope (Axiovert 100, Carl Zeiss, Jena, Germany). The fluorescent signals were corrected for variations in the dye concentration by normalizing the fluorescence (F) against the baseline fluorescence (F_0_) to obtain reliable information about intracellular (cytoplasm) Ca^2+^ ([Ca^2+^]_i_) transient changes from baseline values (F/F_0_) and to exclude variations in the fluorescence intensity with different volumes of injected dye. [Ca^2+^]_i_ transients and peak systolic and diastolic [Ca^2+^]_i_ values were measured during 2‐Hz field stimulation with 10‐ms twice‐threshold strength square‐wave pulses.

After achieving steady‐state Ca^2+^ transients with repeated pulses from −40 to 0 mV (1 Hz for 5 sec.), the total amount of charge crossing the membrane sarcoplasmic reticulum (SR) Ca^2+^ content (Ccaff) was estimated by integrating the sodium (Na^+^)–Ca^2+^ exchanger (NCX) current rapid application of 20 mM caffeine during rest with the membrane potential clamped to −40 mV to cause SR Ca^2+^ release 22. The total SR Ca^2+^ content (expressed as mM of cytosol) was determined by use of the equation: SR Ca^2+^ content = [(1 + 0.12)(Ccaff/F × 1000)]/(Cm × 8.31 × 8.44), where Cm = membrane capacitance; F = Faraday's number; cell surface to volume ratio = 8.44 pF/pL 23, 24, 25.

### APs and ionic currents

A whole‐cell patch clamp was used for single isolated cardiomyocytes with an Axopatch 1D amplifier (Axon Instruments, Foster City, CA, USA) at 35 ± 1°C 26. Borosilicate glass electrodes (o.d., 1.8 mm) with tip resistances of 3~5 MΩ were used. Before the formation of the membrane‐pipette seal, the tip potentials were zeroed in Tyrode's solution. Junction potentials between the bath and pipette solution (9 mV) were corrected for AP recordings. APs were recorded in the current‐clamp mode, and ionic currents were measured in the voltage‐clamp mode. The ionic currents and AP were recorded at approximately similar period (3–5 min.) after membrane rupture or perforation by amphotericin B (for I_Ca‐L_) to avoid decay of ion channel activity over time. A small hyperpolarizing step from a holding potential of −50 mV to a test potential of −55 mV for 80 ms was delivered at the beginning of each experiment. The area under the capacitative current was divided by the applied voltage step to obtain the whole‐cell capacitance. The series resistance (R_s_) was compensated by 60~80%. APs were elicited from isolated cardiomyocytes without spontaneous activity at a driven rate of 1 Hz for 20 beats. The resting membrane potential (RMP) was measured during the period between the last repolarization and the onset of the subsequent AP. The AP amplitude (APA) was obtained from the RMP to the peak of AP depolarization. AP durations (APDs) at 90%, 50% and 20% repolarization were, respectively, measured as the APD_90_, APD_50_ and APD_20_. Micropipettes were filled with a solution containing (in mM) KCl 20, K aspartate 110, MgCl_2_ 1, MgATP 5, HEPES 10, EGTA 0.5, LiGTP 0.1 and Na_2_ phosphocreatine 5 (pH 7.2 with KOH) for the AP and potassium currents; containing (in mM) CsCl 130, MgCl_2_ 1, MgATP 5, HEPES 10, EGTA 10, NaGTP 0.1 and Na_2_ phosphocreatine 5 (pH 7.2 with CsOH) for the L‐type Ca^2+^ current (I_Ca‐L_); containing (in mM) NaCl 20, CsCl 110, MgCl_2_ 0.4, CaCl_2_ 1.75, tetraethylammonium 20, 1,2‐bis(2‐aminophenoxy)ethane‐N,N,N′,N′‐tetraacetic acid (BAPTA) 5, glucose 5, MgATP 5 and HEPES 10 (pH of 7.25) for the NCX current; and containing (in mM) potassium gluconate 144, MgCl_2_ 1.15, EGTA 5, HEPES 10 and CaCl_2_ 4.2 (pH 7.25 with KOH) for the apamin‐sensitive small‐conductance Ca^2+^‐activated K^+^ current (I_KAS_).

The I_Ca‐L_ was recorded using 300‐ms pulses from a holding potential of −50 mV to inactivate the T‐type Ca^2+^ current 27, to test potentials that varied between −40 and +60 mV in 10‐mV increments at a frequency of 0.1 Hz using a perforated patch clamp with amphotericin B. In the external solution, NaCl and KCl of the normal Tyrode's solution were respectively replaced with TEACl and CsCl.

The NCX current was obtained as the nickel‐sensitive current by subtracting the current in the presence of 10 mM NiCl_2_ from that of the control. The recording protocol consisted of 300‐ms pulses ranging from −100 to +100 mV from a holding potential of −40 mV at a frequency of 0.1 Hz. The external solution for measuring the NCX contained (in mM): NaCl 140, CaCl_2_ 2, MgCl_2_ 1, HEPES 5 and glucose 10 (pH of 7.4). It was supplemented with strophanthidin (10 μM), nitrendipine (10 μM) and niflumic acid (100 μM).

The transient outward potassium current (I_to_) was studied using a protocol consisting of a 30‐ms pre‐pulse from a holding potential of −80 to −40 mV to inactivate sodium channels followed by a 300‐ms test pulse to +60 mV in 10‐mV increments at a frequency of 0.1 Hz in the presence of 200 μM CdCl_2_ in Ca^2+^‐free normal Tyrode's solution as described previously 28. The I_to_ was measured as the difference between the peak outward current and the steady‐state current 29, and the sustained outward potassium current (I_Ksus_) was evaluated as the difference between the holding current and the end of the steady‐state current.

The ultra‐rapid delayed rectifier potassium current (I_Kur_) was measured as 4‐aminopyridine‐sensitive currents. To record I_Kur_ without contamination by I_to_, I_Kur_ was dissected by 1 mM 4‐aminopyridine with a double‐pulse protocol, consisting of a 100‐ms depolarizing pre‐pulse to +40 mV from a holding potential of −50 mV to inactivate I_to_, followed by 150‐ms voltage steps from −40 to +60 mV in 10‐mV increments at room temperature to provide adequate temporal resolution 30, 31.

The I_KAS_ was recorded using the whole‐cell mode of the patch‐clamp technique. Stability of the total potassium current (I_K_) was monitored with a step‐pulse protocol (with a holding potential of −50 mV and test potentials from −120 mV to +70 mV for 300 ms at a frequency of 0.1 Hz). Once the I_K_ became stable (usually for 5 min.), apamin (100 nM) was applied, and a step‐pulse protocol was performed when the I_K_ reached a steady state. The I_KAS_ was calculated as the difference between the absence and presence of apamin. All experiments were performed at 36°C. The external solution contained (in mM): N‐methylglucamine, 140; KCl, 4; MgCl_2_, 1; glucose, 5; and HEPES, 10 (pH 7.4 with HCl) 32.

### Western blot analysis

Control and colchicine‐treated HL‐1 cells were centrifuged and lysed using radioimmunoprecipitation (RIPA) buffer. The protein concentration was determined with a Bio‐Rad protein assay reagent (Hercules, CA, USA). For the analysis, equal amounts of protein from each sample were separated using 4~16% Tris‐acetate polyacrylamide gradient gel electrophoresis. After electrophoresis, protein samples were transferred onto equilibrated polyvinylidene difluoride membranes (Amersham Biosciences, Buckinghamshire, UK). Blots were probed with primary antibodies against ryanodine receptor type 2 (RyR; Affinity BioReagent, Golden, CO, USA), RyR phosphorylation at S2808 and S2814 (RyR‐2808 and RyR‐2814; Badrilla Leeds, UK), SR Ca^2+^ ATPase (SERCA2a, Santa Cruz Biotechnology, Santa Cruz, CA, USA), NCX (Swant, Bellinzona, Switzerland), Cav1.2 (I_Ca‐L_ subunit; Alomone Labs, Jerusalem, Israel), phospholamban (PLB; Thermo, Rockford, IL, USA), Thr17‐phosphorylated PLB (PLB‐Thr17; Badrilla, Leeds, UK), PLB‐Ser16 (Badrilla), Ca^2+^/calmodulin‐dependent protein kinase II (CaMKII, Abcam, Cambridge, UK), Kv1.4 (Alomone Labs), Kv1.5 (Alomone Labs), Kv4.2 (Abcam) and α‐actin (Sigma‐Aldrich, St. Louis, MO, USA). Blots were reacted with primary antibodies followed by horseradish peroxidase‐conjugated secondary antibodies. Bound antibodies were detected with an enhanced chemiluminescence (ECL) detection system of an ECL Plus Kit (Millipore, Billerica, MA, USA) and analysed with AlphaEaseFC software (Alpha Innotech, San Leandro, CA, USA). Targeted bands were normalized to α‐actin.

### Immunofluorescence microscopy of cardiomyocyte microtubules

HL‐1 cells with and without colchicine (3 nM) or collagen (10 μg/ml), cultured in Lab‐Tek^™^ 4‐well chamber slides (Thermo Scientific, Rochester, NY, USA) for 24 hrs, were fixed in PBS containing 4% paraformaldehyde for 30 min., blocked with 3% bovine serum albumin and incubated in primary antibody used with anti α‐tubulin antibody (Sigma, St Louis, MO, USA) at a dilution of 1:200 for 2 hrs, and washed three times with PBS for 5 min. Cells were then incubated in secondary antibody used with FITC‐conjugated antibody (Life technologies, Paisley, UK) at a dilution of 1:200 for 1 hr. Finally, counterstaining of nuclei was performed with 4′, 6‐diamidino‐2‐phenylindole (DAPI; Sigma, St Louis, MO, USA). The cells were observed and photographed with a Leica TCS SP5 Confocal Spectral Microscope Imaging System (×200, Leica Microsystems, Wetzlar, Germany).

### Statistical analysis

All quantitative data are expressed as the mean ± standard error of the mean (S.E.M.). A paired *t*‐test was used to compare differences between control and colchicine‐treated HL‐1 cells and collagen‐treated HL‐1 cells with or without colchicine. Nominal variables were compared by a Pearson's chi‐square test or Fisher's exact test. A *P* value of <0.05 was considered statistically significant.

## Results

### Effect of colchicine on Ca^2+^ homeostasis and ionic currents in HL‐1 cells

**Figure 1:**
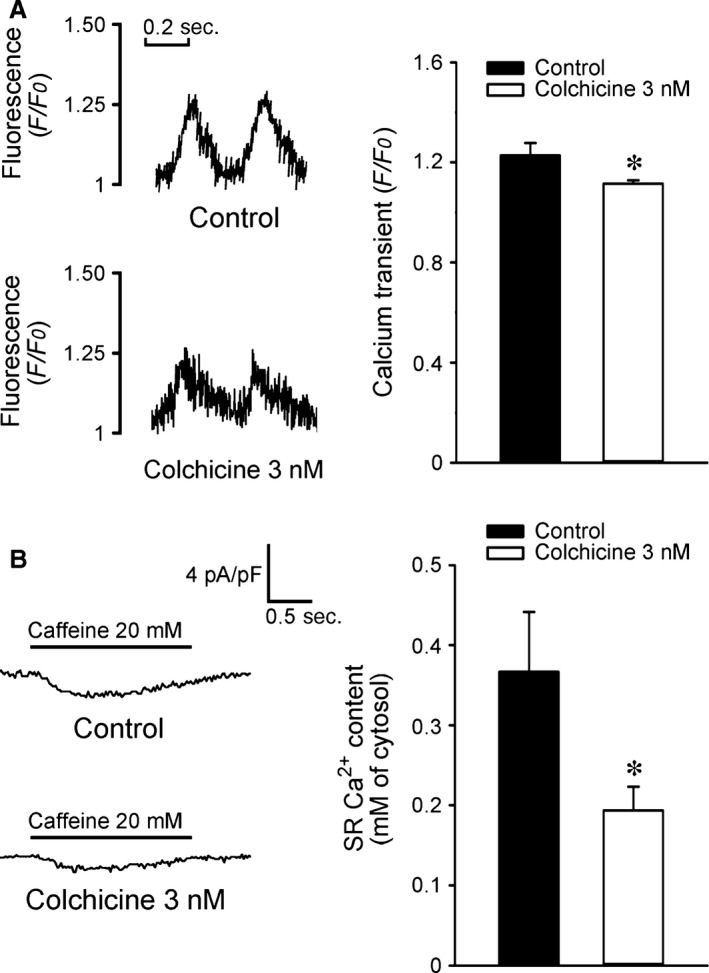
Calcium homeostasis of control and colchicine (3 nM)‐treated HL‐1 cells. (**A**) Tracings and average data from [Ca^2+^]_i_ transients in control (*n* = 16) and colchicine (3 nM)‐treated (*n* = 16) HL‐1 cells. (**B**) Tracings and average data of caffeine‐induced Na^+^–Ca^2+^ exchanger (NCX) currents and sarcoplasmic reticulum Ca^2+^ contents from integrating NCX currents in control (*n* = 12) and colchicine (3 nM)‐treated (*n* = 13) HL‐1 cells. *P < 0.05 *versus* the control.

**Figure 2:**
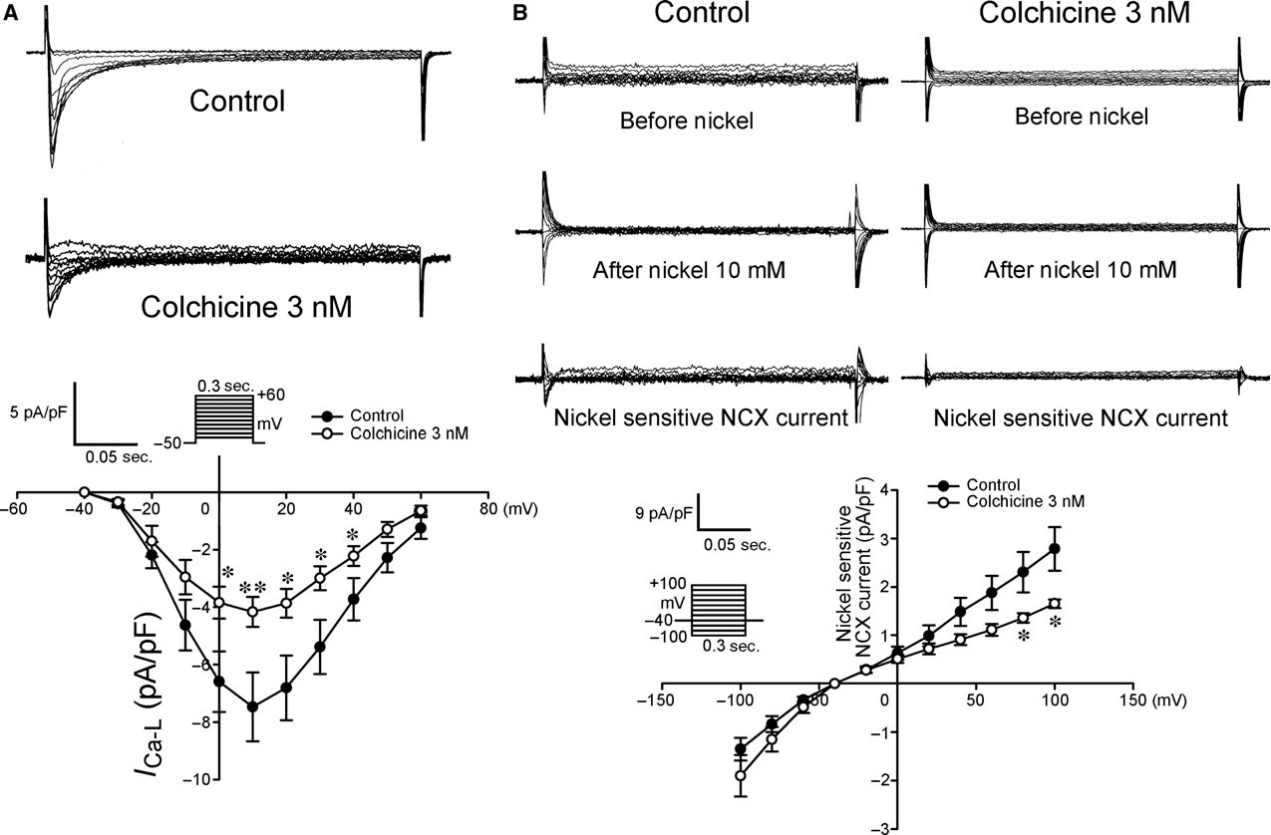
Current tracings and I–V relationship of the L‐type calcium current (I_Ca‐L_) and Na^+^–Ca^2+^ exchanger (NCX) current in HL‐1 cells with and without colchicine (3 nM) treatment. (**A**) The I_Ca‐L_ had decreased amplitudes in colchicine‐treated HL‐1 cells (*n* = 14) than control HL‐1 cells (*n* = 18). (**B**) Colchicine‐treated HL‐1 cells (*n* = 8) exhibited a smaller current amplitude of the NCX current than control HL‐1 cells (*n* = 8). The inset in the current traces shows the clamp protocol.*P < 0.05 *versus* the control, **P < 0.01 *versus* the control.

**Figure 3:**
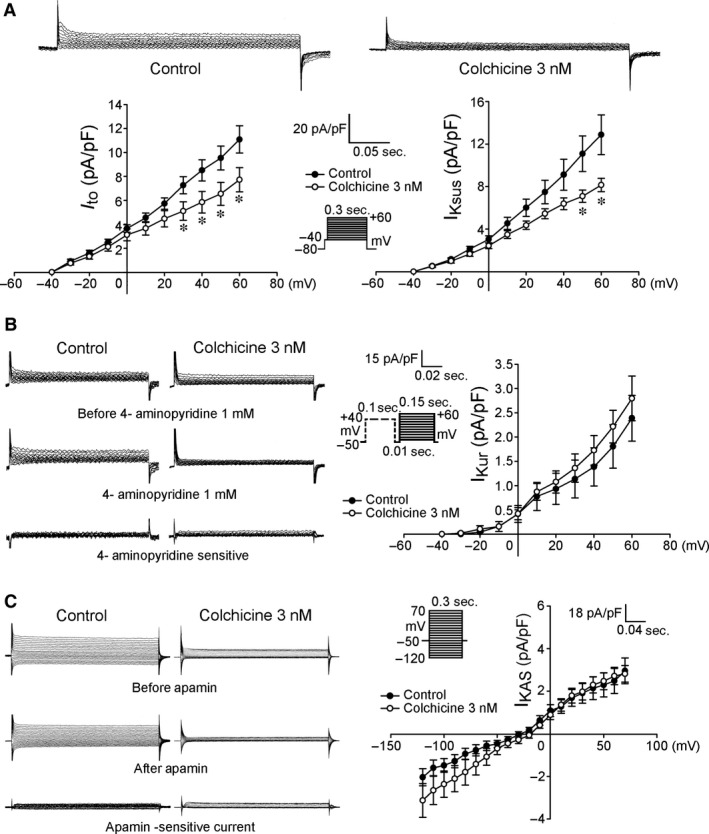
The transient outward potassium current (I_to_), sustained outward potassium current (I_Ksus_), ultra‐rapid delayed rectifier potassium current (I_Kur_) and apamin‐sensitive small‐conductance Ca^2+^‐activated K^+^ current (I_KAS_) in HL‐1 cells with and without colchicine (3 nM) treatment. (**A**) Examples of tracings and I–V relationships of the I_to_ and I_Ksus_ from HL‐1 cells with (*n* = 14) and without (*n* = 15) colchicine treatment. (**B**) Examples of tracings and I–V relationships of the I_Kur_ from HL‐1 cells with (*n* = 8) and without (*n* = 7) colchicine treatment. (**C**) Examples of tracings and I–V relationship of the I_KAS_ from HL‐1 cells with (*n* = 11) and without (*n* = 11) colchicine treatment. Insets in the current traces show the various clamp protocols. *P < 0.05 *versus* the control.

As shown in Figure 1, colchicine (3 nM)‐treated HL‐1 cells had 10% smaller [Ca^2+^]_i_ transients than control HL‐1 cells. Moreover, colchicine (3 nM)‐treated HL‐1 cells had 47% smaller caffeine‐induced [Ca^2+^]_i_ transients than the control group, which suggests less SR Ca^2+^ stores in colchicine (3 nM)‐treated HL‐1 cells. Moreover, colchicine (3 nM)‐treated HL‐1 cells had smaller I_Ca‐L_ and outward (reverse mode) NCX current densities than control HL‐1 cells (Fig. 2). Colchicine (3 nM)‐treated HL‐1 cells had a smaller I_to_ and I_Ksus_ compared with control HL‐1 cells (Fig. 3A). Moreover, colchicine (3 nM)‐treated and control HL‐1 cells had similar values of the I_Kur_ and I_KAS_ (Fig. 3B and C).

### Effects of colchicine on Ca^2+^ regulatory proteins and Kv channel subunits

**Figure 4:**
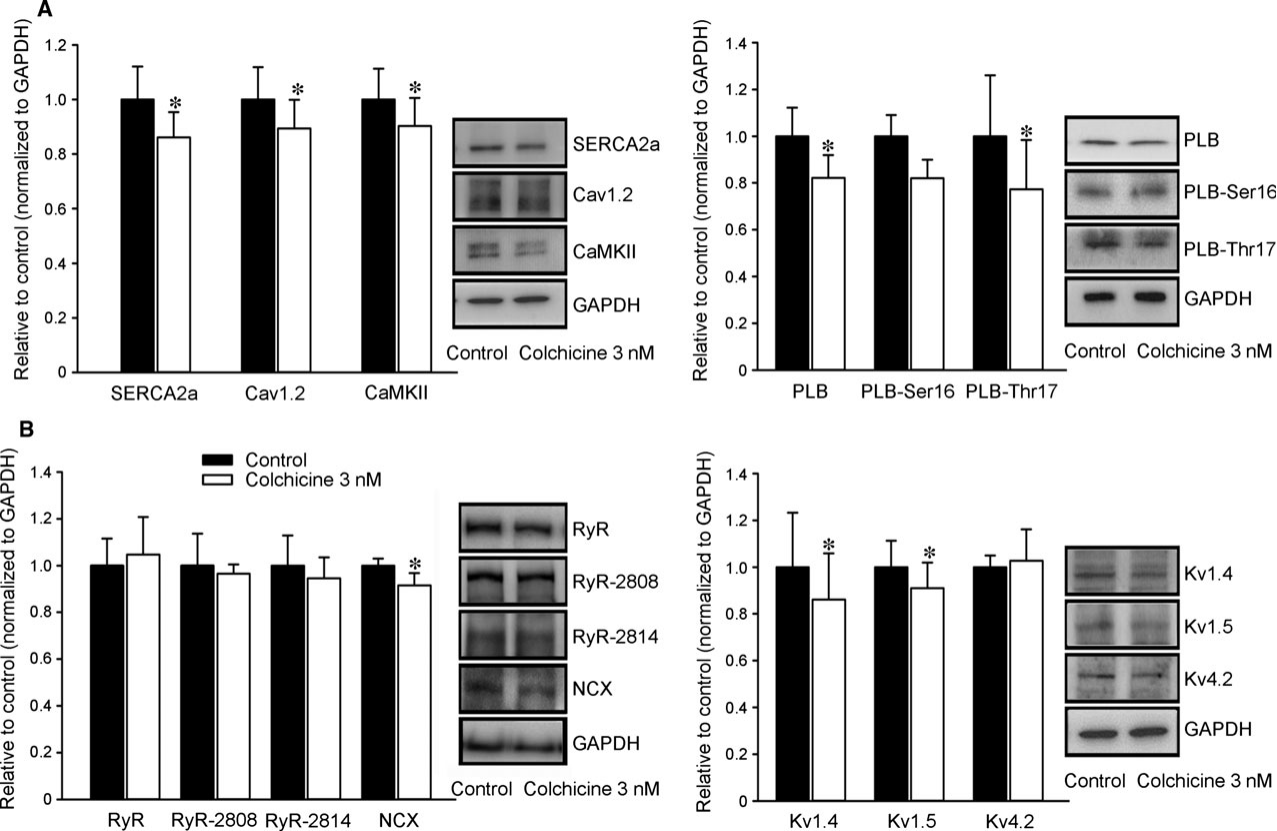
Effect of colchicine on the protein expression of calcium (Ca^2+^) regulatory proteins and potassium channel proteins. (**A**) Representative immunoblotting and average data of sarcoplasmic reticulum Ca^2+^ ATPase (SERCA2a), Cav1.2, Ca^2+^/calmodulin‐dependent protein kinase II (CaMKII), PLB, Ser16‐ and Thr17‐phosphorylated PLB (PLB‐Ser16 and PLB‐Thr17) from control and colchicine (3 nM)‐treated HL‐1 cells. (*n* = 9) (**B**) Representative immunoblotting and average data of RyR type 2, phosphorylation of RyR at S2808 and S2814 (RyR‐2808 and RyR‐2814), the Na^+^–Ca^2+^ exchanger (NCX), Kv1.4, Kv1.5 and Kv4.2 from control and colchicine (3 nM)‐treated HL‐1 cells (*n* = 7). *P < 0.05 *versus* the control. PLB, phospholamban; RyR, ryanodine receptor.

The expression of SERCA2a and Cav1.2 in the colchicine (3 nM)‐treated HL‐1 cells was lower by 14% and 11% than in the controls. Moreover, the expression of CaMKII, a multifunctional serine/threonine protein kinase that mediates Ca^2+^ handling, had also decreased by 15% in colchicine (3 nM)‐treated HL‐1 cells. The colchicine (3 nM)‐treated HL‐1 cells also had lower expressions of total PLB and PLB‐Thr17 by 18% and 23%, but had similar expressions of PLB‐Ser16 (Fig. 4A). The colchicine (3 nM)‐treated HL‐1 cells had lower expression of NCX by 9%, but had similar expressions of RyR, RyR‐2808, and RyR‐2814 compared with control cells. The colchicine (3 nM)‐treated HL‐1 cells had lower expressions of Kv1.4 and Kv1.5 by 10% and 14%, respectively. The expressions of Kv4.2 were similar in both the control and colchicine (3 nM)‐treated HL‐1 cells (Fig. 4B).

### The effect of colchicine on microtubule structure and the AP morphology in collagen‐induced changes of HL‐1 cells

**Figure 5:**
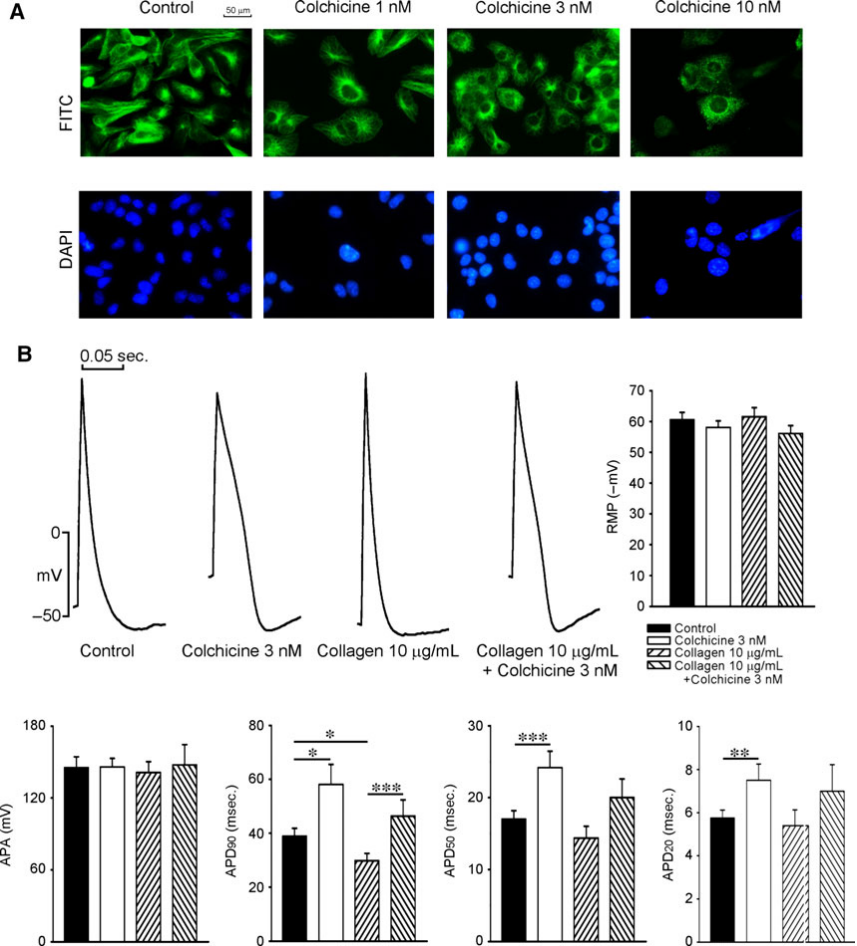
Immunofluorescent staining of microtubules and AP characteristics of HL‐1 cells with and without colchicine or collagen treatment. (**A**) The concentration (1, 3 and 10 nM) effect of colchicine on microtubule structure was studied in HL‐1 cells by confocal microscope. Upper panel shows that HL‐1 cells were labelled with α‐tubulin and then visualized with secondary antibodies conjugated to FITC (green), and lower panel shows that nuclei were stained with DAPI (blue). Colchicine (1 nM) did not have significant effect on microtubule structure, colchicine (3 nM) induced depolymerization with an uneven microtubule structure in HL‐1 cells, and colchicine (10 nM) caused patch aggregations of microtubule in cytoplasm and a decrease in the cell number. (**B**) Action potential (AP) characteristics of HL‐1 cells with and without colchicine (3 nM) or collagen (10 μg/ml) treatment. Examples and average data of APs from control (*n* = 12), colchicine‐treated (*n* = 14), collagen‐treated (*n* = 12) and colchicine and collagen‐treated (*n* = 15) HL‐1 cardiomyocytes. AP durations at 20%, 50% and 90% repolarization of the AP amplitude (APD_20_, APD_50_ and APD_90_, respectively) were measured at 1 Hz. *P < 0.05, **P < 0.01, ***P < 0.005. AP, action potential.

As shown in Figure 5A, a breakdown of the reticular microtubules was evident in the colchicine (3 nM)‐treated HL‐1 cells. When compared with the controls, the colchicine (3 nM)‐treated HL‐1 cells had longer APD_90_, APD_50_ and APD_20_, but had similar values for the APA and RMP. In contrast to the effects of colchicine, collagen (10 μg/ml) shortened the APD_90_ in HL‐1 cells and had more delayed afterdepolarizations (DADs; 20% *versus* 66.7%, *P* < 0.005). Furthermore, colchicine (3 nM) ameliorated APD changes in collagen (10 mg/ml)‐treated HL‐1 cells as shown in Figure 5B, and no DADs were noted.

## Discussion

Ca^2+^ homeostasis is essential for maintaining normal cardiac function and electrical activity. There is tight coupling between Ca^2+^ influx and Ca^2+^ release. Dysregulated Ca^2+^ handling can generate the triggered activity, commonly associated with AF development 33. Ca^2+^ overload may potentially produce arrhythmogenesis in atrial myocytes 34. In this study, colchicine was shown to reduce the amplitude of [Ca^2+^]_i_ transients and SR Ca^2+^ content, which suggests colchicine decreases Ca^2+^ concentration in both cytoplasm and the SR. Accordingly, colchicine might directly modulate Ca^2+^ homeostasis in atrial myocytes and result in a decrease in the occurrence of AF. In contrast, previous studies on rat or chick embryos ventricular myocytes showed that microtubule disruption by colchicine (incubation for 10 min. or 2 hrs, 1~10 μM) increased the I_Ca‐L_ and [Ca^2+^]_i_ transients, but did not modify SR Ca^2+^ content 10, 35, 36, 37. Additionally, other studies showed colchicine had no effect on APD and I_to_ in isolated mouse or dog ventricular myocytes 38, 39. This disparity may arise from different experimental settings (animal species, colchicine concentrations and incubation period) or the variance between HL‐1 cells and ventricular myocytes. As shown in Figure 5A, we have found that colchicine (3 nM) had significant effects on microtubule disruption, which supports the possibility that colchicine may modulate HL‐1 cell calcium homeostasis through microtubule disruption. A previous study showed that patients receiving colchicine had been given therapeutic doses that ranged from 0.5 to 3 μg/l (0.8~7 nM) 20. Therefore, the concentration used in this study was clinically relevant.

In addition to reductions in I_Ca‐L_ and reverse mode NCX in HL‐1 cells, colchicine can down‐regulate SERCA2a, total and PLB‐Thr17. A decrease in PLB phosphorylation at Thr17 can functionally reduce SERCA2a Ca^2+^ reuptake by increasing the effects of PLB on the inhibition of SERCA2a activity. All these changes can result in a decrease in SR Ca^2+^ content and Ca^2+^ transients. Colchicine decreased CaMKII expression, which is a key regulator of excitation‐contraction coupling in cardiomyocytes. CaMKII activation may enhance I_Ca‐L_ facilitation and increase Ca^2+^ influx, which accompanies Ca^2+^ overload. In addition, CaMKII can enhance Ca^2+^ leakage to generate triggered activity. The potential of CaMKII inhibition to reduce an arrhythmogenic outcome was demonstrated in AF 40. This action may result in the known effect of colchicine in decreasing Ca^2+^ sparks from a previous study 10. Therefore, colchicine decreases calcium overload through modulation of Ca^2+^ regulatory proteins, which may inhibit the occurrence of AF.

We found that colchicine can increase the APD, which may reduce the generation of re‐entrant circuits and AF maintenance. This prolongation of the APD may be caused by a reduction of reverse mode NCX, I_to_, and I_Ksus_, which can overcome the APD‐shortening effect by I_Ca‐L_ reduction. Thus, colchicine modulates atrial electrophysiological property by interfering Ca^2+^ and potassium currents, which may ameliorate AF occurrence. Similarly, cytoskeletal disruption was also reported to increase the APD in ventricular myocytes by decreasing the I_to_ 41. These findings suggest that interactions exist between the potassium channels and microtubules or associated cytoskeletal components. The decreased expressions of Kv1.4 and Kv1.5 may contribute to decreases in the I_to_ and I_Ksus_ mediated by colchicine.

The ECM of collagen causes atrial myocytes to exhibit a shorter APD by increasing the I_to_ and I_Ksus_ 34. In this study, it was found that colchicine reversed the APD‐shortening effects of collagen. Moreover, collagen was also shown to induce Ca^2+^ overload, which facilitates the occurrence of trigger activities in atrial and pulmonary vein myocytes. Colchicine normalized the APD in collagen‐treated HL‐1 cells, and this suggests that the ECM controls cytoskeletal mechanics in the development of cardiac remodelling, and colchicine may modulate the remodelling process and atrial arrhythmogenesis.

There are several limitations of our study that must be acknowledged. This study demonstrated the effect of colchicine on microtubule disruption in HL‐1 cells, an atrial cell line from mice. However, the ultrastructure of HL‐1 cells, including the cytoskeleton, and microtubule stability are different from adult naïve cardiomyocytes. 42, 43 Because of the immature phenotype of HL‐1 cells, our findings may not be fully applied to human atrial cells. In addition, we did not study the RyR or SERCA2a activity, and it is not clear whether factors other than those found in this experiment may also contribute to colchicine‐reducing SR content. Sorcin, a penta‐EF‐hand protein, interacts with intracellular target proteins, such as NCX, SERCA2a and I_Ca‐L_ 44, 45, 46, and may influence these current activities. Moreover, pulmonary vein cardiomyocyte plays an important role for triggering AF 47. Ca^2+^ dysregulation may increase arrhythmogenesis in pulmonary vein cardiomyocytes. Colchicine may reduce AF recurrence after pulmonary vein ablation 6. Theoretically, the electrophysiological effects of colchicine on pulmonary vein cardiomyocytes may possibly play a role in its anti‐AF potential.

In conclusion, our findings suggest that colchicine modulates atrial electrical activity and Ca^2+^ regulation, which may be responsible for preventing AF occurrence or recurrence. These changes also demonstrate that microtubule modulation may play an important role in the regulation of ionic channels and Ca^2+^ homeostasis, which contributes to the pathogenesis of AF.

## Conflict of interest

The authors confirm that there are no conflicts of interest.
